# Impact of Christian Orthodox Church Fasting on Metabolic Syndrome Components in Adults Aged 18–49 Years

**DOI:** 10.3390/nu15071755

**Published:** 2023-04-04

**Authors:** Anna Kokkinopoulou, Nikolaos E. Rodopaios, Alexandra-Aikaterini Koulouri, Eleni Vasara, Sousana K. Papadopoulou, Petros Skepastianos, Emmanouil Dermitzakis, Maria Hassapidou, Anthony G. Kafatos

**Affiliations:** 1 Department of Preventive Medicine and Nutrition Unit, School of Medicine, University of Crete, 71003 Heraklion, Greece; kokkinopoulouan@gmail.com (A.K.);; 2 Department of Nutritional Sciences and Dietetics, International Hellenic University, 57400 Thessaloniki, Greece; 3 Laboratory of Animal Physiology, Department of Zoology, School of Biology, Aristotle University of Thessaloniki, 54124 Thessaloniki, Greece; 4 Department of Medical Laboratory Studies, International Hellenic University, 57400 Thessaloniki, Greece; 5 Department of Genetic Medicine and Development, Faculty of Medicine, University of Geneva, 1211 Geneva, Switzerland

**Keywords:** religious fasting, Christian Orthodox Church fasting, Mediterranean Diet, metabolic syndrome

## Abstract

Objective: Studies regarding health effects of religious fasting have been increased during the last decade. Our aim was to investigate the effects of Christian Orthodox Church (COC) fasting on metabolic syndrome in young adults. Methods: Participants were 224 men and women, of whom 111 had been following the COC fasting regime and 113 were non-fasters, all aged 18 to 49 years (mean age 29.23 ± 8.78 years). Anthropometric measurements, including the Bioelectrical Impedance Analysis, were performed on individuals, and they also completed food intake questionnaires, and provided blood samples for biochemical analysis. Metabolic syndrome was defined according to criteria of the National Cholesterol Education Program-Adult Treatment Panel III and all variables were checked. Results: Fasters did not statistically differ in anthropometric measurements when comparing to non-fasters. Differences were found in terms of biochemical variables, and more specific in HDL cholesterol, LDL cholesterol and total blood cholesterol, and in systemic and diastolic blood pressure, although non statistically significant. Statistically significant differences were only noticed in heart pulses and insulin levels, with fasters having lower heart pulses (69.44 ± 9.84 versus 72.63 ± 10.74) and greater mean values of insulin levels (4.16 ± 4.66 versus 3.12 ± 2.35). When analysis was carried to identify which variables were met for metabolic syndrome, it was found that fasters had statistically significant lower values of blood pressure when compared to non-fasters. In more details mean systolic blood pressure was 121.67 ± 12.21 versus 123.41 ± 11.73 for fasters and non-fasters respectively, and mean diastolic blood pressure was 75.77 ± 8.82 versus 78.27 ± 10.07 for fasters and non-fasters. Furthermore, the mean energy intake was higher in non-fasters (1698.25 ± 515.99 kcals) when compared to fasters (1590.24 ± 404.19 kcals) but not statistically significant different. Conclusions: Young adults aged 18 to 49 years who fast according to the COC fasting regimes do not have different metabolic syndrome prevalence when comparing to non-fasters, but there was a statistically significant difference in the prevalence of elevated blood pressure with fasters having lower values.

## 1. Introduction

Fasting, the voluntary abstention from prohibited foods, is a feature of many religions worldwide [1]. The Christian Orthodox Church (COC) has unique dietary recommendations demonstrating an infrequent interchange from a mixed to a vegetarian diet that includes fish and seafood [2]. A fundamental characteristic of COC fasting recommendations is the refrain from meat, dairy products, and eggs for 180–200 days annually while seafoods and snails are allowed in all fasting days in addition to the increased consumption of cereals, legumes, fruits, and vegetables. Fasting occurs at the following periods during the year; 40 days before Christmas (Nativity fast), 48 days before Easter (Lent fast), 15 days before Assumption (Assumption fast), 0 to 30 days prior to the feast of Holy Apostles (depending on Easter feast), three other daily feasts and almost every Wednesday and Friday [2].

During the last decades, an increased interest for the effects of fasting and most specific religious fasting on human health has been recorded in scientific databases, with the majority of the population (84%) identifying as belonging to a religious group, and Christians contribute to the largest group worldwide [3]. According to the most recent reviews of religious fasting, the prudent dietary pattern that COC fasters follow contributes to disease prevention and a sustainable healthy planet [2,4]. Additionally, in individuals with impaired levels in lipid profiles it is shown that other religious fasting, like Ramadan, improves the lipid values [5].

The case of periodic fasting followed by Orthodox Christians in Greece during all COC fasting periods characterizes the traditional Greek diet [6]. The Seven Countries Study (SCS) started in 1960 with 9063 men 40 to 60 years old participating in the study and were followed up for 60 years until extinction. The population of Crete had the highest age of death and most survivors over 100 years old, in comparison to other participating countries, as showed by the most recent publications of SCS [7,8]. This is related to the lifestyle of the population of Crete where more than 60% of men strictly followed all the fasting periods according to COC recommendations, suggesting that the COC fasting is a well-structured prudent dietary pattern that promotes the value of a plant-based diet [9]. As a result of those findings the diet of Crete was named Mediterranean diet although there is huge difference from other Mediterranean countries, with the increased consumption of olive oil, fish, sea food and snails, alongside the reported lowered consumption of meat [10,11,12]. The Ikaria study of the Blue Zones still highlights the importance of the COC diet and showed that high adherence to the Mediterranean diet that follows the same recommendations as the COC fasting diet is linked to lower rates of hypertension, cardiovascular diseases and promotes longevity [13,14,15].

Metabolic syndrome (Mets) is a major public health issue characterized by the co-occurrence of risk factors that includes abdominal obesity, hyperglycemia, dyslipidemia, and hypertension. There are several criteria proposed for the definition of metabolic syndrome, with the most widely accepted criteria those of the National Cholesterol Education Program-Adult Treatment Panel III (NCEP-ATP III) which are in agreement with those of different organizations [16]. Prevalence of metabolic syndrome is increasingly rapidly throughout the world, and having it increases the risk of developing cardiovascular diseases twofold and type 2 diabetes mellitus fivefold [16]. According to a recent review from our team it was shown that COC fasting dietary pattern or its most beneficial components together with physical activity could be advised for the prevention of the metabolic syndrome [2].

To our knowledge, little is known about the prevalence of metabolic syndrome in young adults COC fasters. Therefore, the aim in undertaking the present study was to investigate the prevalence of metabolic syndrome and the relationship with COC fasting status in a Greek population for the first time.

## 2. Methods

### 2.1. Population Sample

This is part of a larger cross-sectional study, conducted in Thessaloniki, Greece, [17,18] and aimed at examining the effects of COC fasting on health, including obesity, hypertension, dyslipidemia, hyperglycemia, and metabolic syndrome, among others, by comparing individuals who follow the COC fasting versus individuals who do not follow any specific dietary pattern. Thus, 400 participants were chosen, equally divided according to their diet status, gender and two age groups; 18–49 and over 50 years old.

Participants were recruited on a voluntary basis through public Universities, churches and monasteries in the prefecture of Thessaloniki. All participants provided written informed consent to participate in the study, after reading the participant information sheet and understanding the purpose of the study, and were free to withdraw at any time. The study protocol was approved by the Bioethics Committee of the Alexander Technological Educational Institute of Thessaloniki. Volunteers were all screened via a small initial questionnaire with closed-ended question regarding diet status; those qualified as fasters have been following the COC fasting regime since their childhood (and more specific for those who were 18 years old had to follow the COC fasting since the age of 10 years), and those qualified as non-fasters had to declare no avoidance of any food(s) for any medical or other reason, like following a lacto-ovo-vegetarian diet.

Inclusion criteria were to be an adult, be able to provide written informed consent, be able to attend the scheduled appointment and participate in all anthropometric measurements and collected data, being in good general health with no long-term conditions, no current use of medication and/or dietary supplementation. Overall, 454 individuals expressed their wish to participate in the study, but those who did not meet the inclusion criteria were excluded from attending a related appointment with the researchers.

In this study we were focused on results in the younger group aged 18.0 to 49.9 years old. Two hundred and twenty-four individuals (81 men, 143 women), mean age 29.23 ± 8.78 years, participated in the study. Out of them, one hundred eleven individuals (38 men, 73 women), mean age 30.05 ± 8.88 years, fasted regularly according to the fasting recommendations of COC since their childhood, for a mean 25 years of fasting starting at the age of 10 years. Another group of 113 individuals (43 men, 70 women), mean age 28.41 ± 8.94 years, were control subjects that did not fast.

As described, adherence to the COC fasting regime includes abstaining from foods of animal origin, with the exception of seafood and snails, during five periods of the year accounting for a total of a maximum 200 days, depending on when Easter is feasted each year. Therefore, to ensure that data regarding fasting population was as representative of the whole year as possible, measurements were not performed during a fasting day and/or period; instead, blood sampling and all measurements were collected on Saturdays during a period when fasting was on Wednesdays and Fridays.

### 2.2. Prevalence of Metabolic Syndrome

Metabolic syndrome has become a public health concern and, as defined by the World Health Organization (WHO), is a cluster of disorders that includes abdominal obesity, insulin resistance, dyslipidaemia and hypertension [19]. Based on the NCE-ATP III definition, metabolic syndrome is presence when 3 or more of the following risk factors are present; waist circumference > 102 cm in men or >88 cm for women, fasting blood glucose ≥ 100 mg/dL or treated with low-glucose medication, high-density lipoprotein (HDL) cholesterol < 40 mg/dL in men or <50 mg/dL in women or treated with lipid lowering medication, blood triglyceride levels ≥ 150 mg/dL or treated with lipid lowering medication and blood pressure levels ≥ 130/85 mmHg or treated with hypertensive medication [20].

### 2.3. Anthropometric and Hematological Measurements

An experienced dietitian performed all anthropometric measurements in the morning, with participants in minimal clothing. Height was measured to the nearest 0.5 cm using a stadiometer, with an accuracy of 0.5 cm. Body weight was measured with the use of a calibrated digital scale with an accuracy of ±100 g. Body Mass Index (BMI) was calculated as body weight divided by the square of height (kg/m^2^). Waist and hip circumference were measured with a tape over the naked skin. Waist to Hip Ratio (WHR) was defined as waist circumference (cm)/hip circumference (cm), with results ≥1.0 and ≥0.85 indicating obesity for males and females respectively. The Bioelectrical Impedance Analysis (BIA) method was used to assess body fat, fat free mass, muscle mass and total body water with the use of a calibrated Bioimpedance Analyser. Last, bone health parameters, including bone mineral density and bone mineral content, were evaluated using dual-energy X-ray absorptiometry (Lunar DPX Bravo, GE Healthcare, Chicago, IL, USA).

All measurements were taken in the morning, with participants fasting after midnight on the day before the appointment. Last, blood pressure (BP) and resting heart rate were measured with an electronic blood pressure monitor (Omron, Hoffman Estates, IL, USA) in sitting positions after resting for 10 min, and 6 mL of venous blood was collected to measure biochemical parameters [17,18].

### 2.4. Socioeconomic and Lifestyle Habits

All subjects completed a validated questionnaire regarding their socio-economic status (educational level, marital status) and lifestyle habits, with questions including, physical activity, smoking status, time spending using the computer, time watching television, and time sleeping, among others. The combination of all the above mentioned questionnaires was used in a previous randomized controlled nutritional intervention study in Greece [21].

### 2.5. Statistical Analysis

Statistical analysis was performed with the SPSS version21 software (SPSS, Chicago, IL, USA). Continuous data are shown as means with standard deviation (SD) and categorical variables as relative frequencies with percentages. The chi-squared test was used to test for differences among categorical variables, while Student’s *t*-test and One Way Analysis of Variance (ANOVA) were used to test for differences in continuous variables among two or more groups, respectively. All data were normally distributed. Statistical significance was set at *p* = 0.05.

## 3. Results

Eighty-one men and 143 women participated in the study, with a mean age of 29.23 ± 8.78 years, mean weight 71.31 ± 16.48 kg and mean BMI 24.78 ± 4.31 kg/m^2^. Mean waist circumference was 83.97 ± 13.27 cm, mean hip circumference was 97.56 ± 8.36 cm and mean waist to hip ratio was 0.86 ± 0.10. When analysis was carried based on sex, it was found that men had statistically significant higher values in body weight (*p* < 0.001), body height (*p* < 0.001), BMI (*p* < 0.001), fat free mass (*p* < 0.001) and waist circumference (*p* < 0.001), while women had statistically significant higher mean values in body fat (*p* < 0.001).

The majority of the sample had tertiary education (66.1%), most of the participants were single (75%), they never smoked (81.7%) and among smokers only a few (35.2%) smoked more than 10 cigarettes per day.

Comparative analysis by fasters and non-fasters groups can be seen in Table 1. The Chi-Square results show that there is a statistically significant relationship between fasting status and marital status (*p* = 0.011) and smoking habits (*p* < 0.001). In more details, 32.4% fasters were married when comparing to 17.7% non-fasters who did the same. Additionally, only a small percentage of fasters smoked (8.1%) compared to almost one fourth (24.8%) of non-fasters. No further statistically significant differences were found based on gender (*p* = 0.55), education level (*p* = 0.26), BMI status (*p* = 0.40) and physical activity status (*p* = 0.27).

In regards to anthropometric variables, there were small differences in the mean values between fasters and non-fasters, but they were not statistically significant. In more details, fasters had lower mean energy intake when comparing to non-fasters (1590.24 ± 404.19 vs. 1698.25 ± 515.99 respectively, *p* = 0.083), as well as fasters had lower mean values of systolic and diastolic blood pressure, fasting blood glucose, HDL cholesterol, LDL cholesterol and total cholesterol (*p* = 0.27, *p* = 0.053, *p* = 0.13, *p* = 0.99, *p* = 0.35, *p* = 0.09 respectively). Furthermore, when looking to the biochemical parameters we noticed a statistically significant difference in the pulses with non-fasters having higher mean values when comparing to fasters (72.63 ± 10.74 vs. 69.44 ± 9.84 respectively, *p* = 0.022), and in the insulin levels with fasters having greater mean values compared to non-fasters (4.16 ± 4.66 vs. 3.12 ± 2.35 respectively, *p* = 0.036).

When investigating the metabolic syndrome prevalence, all components contributing to metabolic syndrome were checked. In more details, waist circumference was found to be over the cut offs (102 cm for men and 88 cm for women) in 12 men and 36 women of both groups, and HDL was seen below the cut offs (40 mg/dL for men and 50 mg/dL for women) in 21 and 37 men and women respectively. Fasting blood glucose was above the 100 mg/dL cut-off in two fasters and eight non-fasters, triglyceride levels were above 150 mg/dL for 31 fasters and 28 non-fasters, while increased blood pressure (above 130/85 mmHg) was seen in 73 fasters and 11 non-fasters. Available data is shown in Table 2.

No components for metabolic syndrome had 41.4% fasters and 41.6% non-fasters, while one component was seen in 34 fasters (30.6%) and 35 non-fasters (31%), and two components were found in 20 fasters as well as 20 non-fasters (18% and 17.7% respectively). Three components of metabolic syndrome were found in 11 fasters (9.9%) and nine non-fasters (8%), four components in no fasters and two non-fasters (1.8%), while five components were not found in any of the two groups. In addition, there was no statistically significant difference in the number of components when comparing the two groups (*p* = 0.945). Available data for metabolic syndrome components is shown in Table 3.

When analysis was carried based on fasting status, it was found that there was no statistically significant difference in metabolic syndrome prevalence (*p* = 0.96). When looking the components accounting for metabolic syndrome independent, it was shown that only blood pressure was statistically significant different with non-fasters having higher mean values of systolic and diastolic blood pressure when comparing to fasters (*p* = 0.020). No statistically difference was found in waist status (*p* = 0.09), fasting blood glucose (*p* = 0.056), HDL cholesterol (*p* = 0.49) and triglycerides (*p* = 0.59).

When analysis was based on gender in the group of fasters, it was shown that there were some statistically significant differences in anthropometric and biochemical data. Women had statistically significant higher mean values in body fat (*p* < 0.001), HDL cholesterol (*p* < 0.001) and in glucose levels (*p* = 0.015). On the other hand, men had statistically significant higher mean values in body weight (*p* < 0.001), body height (*p* < 0.001), BMI (*p* = 0.025), fat free mass (*p* < 0.001), waist circumference (*p* = 0.011), waist to hip ratio (*p* = 0.031), systolic and diastolic blood pressure (both with *p* < 0.001), creatinine (*p* < 0.001), uric acid (*p* < 0.001), albumin (*p* < 0.001), γ-GT (*p* = 0.024), triglycerides (*p* = 0.040), SGOT (*p* = 0.042) and glucose (*p* = 0.001). In regards to details of the components that contribute to metabolic syndrome, significantly higher values in blood pressure were noticed in men (*p* = 0.002), while in the rest components no significantly difference was noticed (waist circumference *p* = 0.18, HDL *p* = 0.86, triglyceride levels *p* = 0.051 and glucose levels *p* = 0.63.

In the group of non-fasters, when analysis was based on gender, results showed that statistically significant differences were found in anthropometric measurements and biochemical variables. Women had significantly higher mean values in body fat (*p* < 0.001) and HDL cholesterol (*p* < 0.001). Men had significantly higher mean values in body weight (*p* < 0.001), body height (*p* < 0.001), BMI (*p* = 0.001), fat free mass (*p* < 0.001), waist circumference (*p* < 0.001), hip circumference (*p* < 0.001 waist to hip ratio (*p* < 0.001), systolic and diastolic blood pressure (both *p* < 0.001), iron (*p* = 0.041), creatinine (*p* < 0.001), urea (*p* < 0.001), uric acid (*p* < 0.001), (*p* < 0.001), triglycerides (*p* = 0.032), SGPT (*p* = 0.023), and magnesium (*p* = 0.007). With analysis focusing on the different variables of the metabolic syndrome it was noticed that men had statistically significantly higher mean values in triglyceride levels (*p* = 0.004), elevated mean values in blood pressure (*p* < 0.001) and met significantly more metabolic syndrome components (*p* = 0.008) when comparing to women. In the rest of the components no statistically significant differences were noticed (waist circumference *p* = 0.25, HDL *p* = 0.90 and glucose level *p* = 0.47).

Further analysis regarding metabolic syndrome prevalence has been made to investigate other correlations among socio-economic factors, with ANOVA showing no significant differences between metabolic syndrome presence and education, family, smoking and alcohol status (*p* = 0.83, *p* = 0.07, *p* = 0.40, and *p* = 0.26 respectively). Statistically significant differences were found between metabolic syndrome presence and BMI status (*p* < 0.001) with those being overweight or obesity having metabolic syndrome, and between physical activity level (*p* < 0.001) with those having reporting doing physical activity never/rarely or <2 times per week having metabolic syndrome.

Further analysis of the data with the use of general linear model showed that the BMI status and the gender were highly significant for the presence of metabolic syndrome components (*p* < 0.001 and *p* = 0.026 respectively). The starting model included gender, age, physical activity, BMI, smoking and family status with the final adjusted model to include only gender and BMI status. It was shown that the metabolic syndrome components were decreased with low BMI values, while the metabolic syndrome components were increased with the presence of male participants.

A logistic regression model was used to build a model to predict the probability of metabolic syndrome prevalence, using fasting status, gender, age, physical activity, smoking and family status as predictors. The classification accuracy was 90.2%. The variables included in the equation, showed that there was a negative association with the gender of participants, with men meeting less components for metabolic syndrome (*p* = 0.02). There was a negative association with physical activity, with individuals having increased levels of physical activity not having metabolic syndrome (*p* < 0.001). Results can be seen in Table 4. Furthermore, logistic regression analysis was undertaken individually for all components contributing to metabolic syndrome. In more details, for the waist status variable a positive association with the age was found, and more specifically while age was increased the waist status values were elevated (*p* < 0.001). BMI status was positive correlated, with those having higher values of BMI having also higher values of waist circumference (*p* = 0.005). Regarding the fasting blood glucose levels, positive correlation was seen with the diet status, with higher values in non-faster individuals (*p* = 0.024). To continue with, lower levels of HDL cholesterol showed no associations with the variables. Elevated triglyceride levels were negatively associated with gender, meaning that males had lower mean values of triglycerides (*p* = 0.001). Last, increased blood pressure was positively associated with diet status, with non-fasters having higher mean blood pressure values (*p* = 0.042), while gender was negatively associated with males having lower levels of blood pressure (*p* < 0.001) (details can be seen in Table 5).

## 4. Discussion

To the best of our knowledge, this is the first study to investigate the prevalence of metabolic syndrome in a COC fasting population in Greece. A limited number of studies have examined anthropometric and biochemical parameters in fasting and non-fasting periods [22,23,24,25,26,27], while according to a recent scoping review no study has presented data focusing on metabolic syndrome [2].

According to our results, metabolic syndrome does not differentiate in young adults who follow a COC fasting regime, with 9.9% and 9.7% of fasters and non-fasters having metabolic syndrome in the study population. In terms of gender no statistically significant differences were found when analysis was carried.

In our study when analysis was carried to identify which variables were met for metabolic syndrome, it was found that a statistically significant lower number of fasters had elevated blood pressure (a major component of metabolic syndrome) when comparing to non-fasters. This is a similar finding to the study of Sarri and colleagues with 38 COC fasters in Crete, that reported no difference in blood pressure of fasters when comparing to non-fasters [28]. The reason could be probably the high salt intake by both groups, as already mentioned in the studies, but this has to be investigated thoroughly in a future study, where nutrient intake is taken into account. Elsayed and colleagues reported that, after an Easter fasting period, fasters with and without T2DM decreased systolic blood pressure and increased diastolic blood pressure, and fasters with hypertension lowered both systolic blood pressure and diastolic blood pressure after the fasting period [24]. Our study showed that fasters had lower diastolic blood pressure, similar to a study with 100 adults who were above 50 years old and followed the COC fasting regime that found lower diastolic blood pressure levels when comparing to a control group of 100 non-fasters [18]. Similarly, in other studies in a Greek population, the results of the EPIC study indicated that adherence to the Mediterranean diet is inversely associated with blood pressure levels [29] and the ATTICA study showed that a food pattern characterized by consumption of fish, vegetables, fruits, legumes and cereals was inversely associated with blood pressure [30]. Significant reduction in diastolic blood pressure and lower odds of hypertension was also observed in 302 vegetarians and vegans participated in the Adventist Health Study-2 (AHS-2) when comparing to 198 non-vegetarians [31]. According to recent reviews, it is shown that plant-based and/or vegetarian diets are associated with lowering blood pressure levels when comparing to omnivorous diets [32,33].

A high percentage of participants were with overweight and obesity in both fasting and non-fasting population of our study (43.35% and 37.82% respectively). This could be a result of increased energy intake and/or of abstinence of self-discipline in the national dietary guidelines and the recommended portion sizes alongside a reduced physical activity pattern. The results are similar to a recent study in COC fasters in Greece that revealed that the majority of 100 faster and 100 non-faster participants were overweight and obese [34]. In a study of Ching and colleagues with 273 vegetarians in Malaysia it was found that approximately half of the participants were with overweight and obesity [35].

Regarding our biochemical data, it was shown that fasters had lower mean values of total cholesterol, HDL and LDL levels when compared to non-fasters, but not statistically significant. Basilakis and colleagues showed that 36 fasters, that were also members of five different Monasteries in Greece, had lower levels of cholesterol, HDL and LDL after the Christmas fasting period [36]. The same reduction in total cholesterol and LDL levels was seen after the Christmas fasting period in a population of 37 fasters in Crete, as reported by Sarri and colleagues [27]. Additionally, Papadaki and colleagues showed that, after an Easter fasting week, there were lower levels of total cholesterol and LDL as a result of increased fruit, vegetable, legume, fish, shellfish, snail and nut consumption [37]. In a study with a focus on the three major fasting periods in Greece it was shown that fasters had 12.5% lower total cholesterol and 15.9% lower LDL when comparing to non-fasters, another finding that comes in accordance with our findings [38]. Decreased cholesterol levels after a fasting period have been reported in 49 COC fasters living in Egypt [26] and in 99 COC fasters living in the USA [22]. Similar results were shown in the PREDIMED study, where participants with higher adherence to the Mediterranean diet had 47% lower odds of having low HDL levels [39]. The EPIC-Oxford study, with 11,004 participants in the UK, showed that mean LDL cholesterol was 12% lower in vegetarians than in omnivores [40]. The ATTICA study in Greece, with 3042 participants, showed that the Mediterranean diet was positively associated with HDL levels [30].One interesting result was that fasters seemed to follow a healthier way of lifestyle, with 91.9% of them being non-smokers, when comparing to 75.2% of non-fasters. This comes to an agreement with a study in Greece that revealed that COC fasting is associated with positive health related behaviors, like absence from smoke and alcohol [41]. Similar lifestyle habits were noticed in Malaysian religious vegetarians were 95.2% of them were non-smokers [35].

The present study is a first for metabolic syndrome prevalence in COC fasters and could be used as a base for future research. Another strength of this cross-sectional study is the large number of participants in the study, with the total number of 113 fasters according to COC recommendations and 111 non-fasters meeting the acceptable sample size for proper statistical analysis and reliable results. Misreporting of any self-reported variable, including smoking status, nutrient intake and other variables is considered as a limitation of the study.

## 5. Conclusions

The findings of the present study suggest that metabolic syndrome prevalence is not statistically different based on the diet status of young adults aged 18 to 49 years. Fasters of young age have been found to have lower values of HDL cholesterol, LDL cholesterol and total cholesterol, when comparing to non-fasters. It was also found that a statistically significant lower number of fasters had elevated blood pressure when comparing to non-fasters. COC fasting recommendations could be followed by the general population who wish to follow a plant-based diet in terms of a healthier way of living, as there is no deficiency when checking the biochemical parameters, and should always be followed under personalized guidance on proper meal planning, like any other dietary pattern. Future analysis should investigate the difference in an older age group, i.e., above 50 years.

Furthermore, differences in the anthropometric, biochemical and cardiac parameters have to be addressed thoroughly through nutrient and energy intake analysis, given also the high prevalence of overweight and obesity in both groups. Future studies could be also focused on monitoring a fasting sample for five or ten years and analyzing different health aspects, like cardiovascular diseases and metabolic syndrome, in order to establish conclusive results.

## Figures and Tables

**Table 1:** Comparative analysis by group.

Variable	Fasters (n = 111)		Non-Fasters (n = 113)		
	N	%	N	%	*p*-Value
**Gender**					0.55
Male	38	34.2	43	38.1	
Female	73	65.8	70	61.9	
**Education level**					0.26
Primary education	0	0	2	1.8	
Secondary education	18	16.2	24	21.2	
Tertiary education	79	71.2	69	61.1	
Master’s/Doctoral	14	12.6	18	15.9	
**Family status**					0.011
Single	75	67.6	93	82.3	
Married/Living together	36	32.4	20	17.7	
**Smoking status**					0.000
Yes	9	8.1	28	24.8	
No—never	98	88.3	85	75.2	
No—quit smoking	4	3.6	0	0	
**BMI status**					0.40
Underweight	0	0	2	1.8	
Normal weight	62	54.86	69	62.16	
Overweight	37	32.74	32	28.82	
Obesity	12	10.61	10	9.0	
**Physical activity status**					0.27
Very low (never/rarely)	8	7.2	12	10.6	
Low (<2 times per week)	24	21.6	28	24.8	
Moderate (2–3 times per week)	59	53.2	58	51.3	
High (3–5 times per week)	18	16.2	11	9.7	
Professional athlete (everyday_	2	1.8	4	3.5	
	**Mean**	**SD**	**Mean**	**SD**	***p*-value**
**Age (years)**	30.05	8.58	28.41	8.94	0.55
**Weight (kg)**	71.60	16.43	71.04	16.59	0.80
**Height (m)**	1.68	0.08	1.69	0.09	0.15
**BMI (kg/m^2^)**	25.15	4.51	24.40	4.08	0.19
**Fat mass (kg)**	20.54	10.37	19.05	8.90	0.25
**Waist circumference (cm)**	84.17	13.86	83.77	12.71	0.82
**Hip circumference (cm)**	97.03	9.30	98.08	7.32	0.34
**WHR**	0.86	0.11	0.85	0.09	0.25
**Energy intake (kcal)**	1590.24	404.19	1698.25	515.99	0.08
**Systolic blood pressure (mmHg)**	121.67	12.21	123.41	11.73	0.27
**Diastolic blood pressure (mmHg)**	75.77	8.82	78.24	10.07	0.053
**Pulses**	69.44	9.84	72.63	10.74	0.022
**Glucose (mg/dL)**	78.16	10.88	80.69	14.08	0.13
**Triglycerides (mg/dL)**	125.32	89.40	119.67	64.10	0.58
**HDL (mg/dL)**	54.58	14.29	54.59	14.42	0.99
**LDL (mg/dL)**	98.16	58.18	105.12	54.56	0.35
**Insulin (μIU/mL)**	4.16	4.66	3.12	2.35	0.036
**Cholesterol (mg/dL)**	169.95	36.40	178.34	39.10	0.09

**Table 2:** Prevalence of metabolic syndrome and its components.

Variable	Fasters (n = 111)	Non-Fasters (n = 113)	*p*-Value
**WC > 102 cm for men or** **>88 cm for women, n (%)**	29 (26.1)	19 (16.8)	0.09
**FBG** **≥ 100 mg/dL, n (%)**	2 (1.8)	8 (7.1)	0.49
**HDL cholesterol < 40 mg/dL for men or <50 mg/dL for women, n (%)**	31 (27.9)	27 (23.9)	0.59
**TRG** **≥ 150 mg/dL, n (%)**	31 (27.9)	28 (24.8)	0.056
**BP** **≥ 130/85 mmHg, n (%)**	14 (12.6)	28 (24.8)	0.020
**Metabolic syndrome, n (%)**	11 (9.9)	11 (9.7)	0.96

WC: Waist Circumference, FBG: Fasting Blood Glucose, HDL: High-Density Lipoprotein, TRG: Triglycerides, BP: Blood Pressure.

**Table 3:** Metabolic syndrome components of the participants.

Components of Metabolic Syndrome	Fasters			Non-Fasters		
	All (n = 111)	Men (n = 38)	Women (n = 73)	All (n = 113)	Men (n = 43)	Women (n = 70)
**0, n (%)**	46 (41.4)	13 (34.2)	33 (45.2)	47 (41.6)	11 (25.6)	36 (51.4)
**1, n (%)**	34 (30.6)	10 (26.3)	24 (32.9)	35 (31.0)	16 (37.2)	19 (27.1)
**2, n (%)**	20 (18.0)	11 (28.9)	9 (12.3)	20 (17.7)	9 (20.9)	11 (15.7)
**3, n (%)**	11 (9.9)	4 (10.5)	7 (9.6)	9 (8.0)	6 (14.0)	3 (4.3)
**4, n (%)**	0	0	0	2 (1.8)	1 (2.3)	1 (1.4)
**5, n (%)**	0	0	0	0	0	0
**Metabolic syndrome prevalence, n (%)**	11 (9.9)	4 (10.5)	7 (9.6)	11 (9.7)	7 (16.3)	4 (5.7)

**Table 4:** Logistic regression analysis results.

Variables in the Equation							
		B	S.E.	Wald	df	Sig.	Exp(B)
Step 1 ^a^	Fasting_status	−0.149	0.499	0.090	1	0.765	0.861
	Gender	−1.165	0.510	5.208	1	0.022	0.312
	Age	0.066	0.034	3.749	1	0.053	1.068
	Education_status	0.397	0.400	0.989	1	0.320	1.488
	Family_status	−0.218	0.688	0.101	1	0.751	0.804
	Smoking_status	0.275	0.491	0.314	1	0.575	1.317
	PA_status	−1.062	0.292	13.209	1	0.000	0.346
	Constant	−0.526	2.297	0.052	1	0.819	0.591

^a^ Variable(s) entered on step 1: Fasting_status, Gender, Age, Education_status, Family_status, Smoking_status, PA_status.

**Table 5:** Logistic regression analysis results for blood pressure.

Variables in the Equation							
		B	S.E.	Wald	df	Sig.	Exp(B)
Step 1 ^a^	Fasting_status	0.810	0.398	4.138	1	0.042	2.248
	Gender	−1.931	0.400	23.260	1	0.000	0.145
	Age	0.024	0.029	0.690	1	0.406	1.024
	Education_status	−0.246	0.314	0.614	1	0.433	0.782
	Family_status	−0.467	0.629	0.551	1	0.458	0.627
	Smoking_status	−0.099	0.440	0.050	1	0.823	0.906
	PA_status	−0.065	0.215	0.091	1	0.763	0.937
	Constant	0.964	1.811	0.283	1	0.594	2.623

^a^ Variable(s) entered on step 1: Fasting_status, Gender, Age, Education_status, Family_status, Smoking_status, PA_status.

## Data Availability

Data available on request due to privacy and ethical restrictions. The data presented in this study are available on request from the corresponding author. The data are not publicly available due to security.
